# Clinical characteristics and survival of Chinese patients diagnosed with pulmonary arterial hypertension who carry *BMPR2* or *EIF2KAK4* variants

**DOI:** 10.1186/s12890-020-01179-7

**Published:** 2020-05-29

**Authors:** Qixian Zeng, Hang Yang, Bingyang Liu, Yanyun Ma, Zhihong Liu, Qianlong Chen, Wenke Li, Qin Luo, Zhihui Zhao, Zhou Zhou, Changming Xiong

**Affiliations:** 1 State Key Laboratory of Cardiovascular Disease, Center of Pulmonary Vascular Disease, Fuwai Hospital, National Center for Cardiovascular Disease, Chinese Academy of Medical Sciences and Peking Union Medical College, North Lishi Road, Xicheng District, No.167, Beijing, China; 2 State Key Laboratory of Cardiovascular Disease, Beijing Key Laboratory for Molecular Diagnostics of Cardiovascular Disease, Diagnostic Laboratory Service, Fuwai Hosptial, National Center for Cardiovascular Diseases, Chinese Academy of Medical Sciences and Peking Union Medical College, North Lishi Road, Xicheng District No.167, Beijing, China

**Keywords:** *BMPR2* variants, Biallelic *EIF2AK4* variants, PVOD/PCH, Survival

## Abstract

**Background:**

Variants in the gene encoding bone morphogenetic protein receptor type II (*BMPR2*) are the most common genetic cause of pulmonary arterial hypertension (PAH), whereas biallelic variants in the eukaryotic translation initiation factor 2 alpha kinase 4 gene (*EIF2AK4*) are described in pulmonary veno-occlusive disease/pulmonary capillary haemangiomatosis (PVOD/PCH). Racial background may influence the clinical characteristics of patients diagnosed with PAH or PVOD/PCH. Here, we compared the clinical characteristics and survival between patients with *BMPR2* variants or *EIF2AK4* variants in a Chinese population.

**Methods:**

Heterozygous variants in *BMPR2* and homozygous or compound heterozygous biallelic *EIF2AK4* variants predicted to be deleterious were identified as potentially causal. Clinical and radiological data were collected and analysed. The primary outcomes were death or lung transplantation. Hazard ratios (HRs) for death or transplantation associated with the presence of *BMPR2* or biallelic *EIF2AK4* variants were calculated using Cox proportional hazards models to analyse patient survival.

**Results:**

Two hundred thirty-two patients with PAH were enrolled for genetic testing, and PAH patients with associated conditions were excluded from the study. Forty-five patients with *BMPR2* variants and 11 patients with biallelic *EIF2AK4* variants were recruited. PAH patients with *BMPR2* or biallelic *EIF2AK4* variants presented symptoms at the ages of 25.57 ± 10.17 years and 31.6 ± 9.38 years, respectively. The whole group of patients showed female dominance either with *BMPR2* variants or biallelic *EIF2AK4* variants. Specific radiological abnormalities are more prominent in *EIF2AK4* variant carriers but can also be found in some patients with *BMPR2* variants. Biallelic *EIF2AK4* variant carriers had worse survival than *BMPR2* variant carriers (*p* < 0.0001).

**Conclusions:**

Clinical pictures of PAH patients with *BMPR2* and biallelic *EIF2AK4* variants in the Chinese population differ from other populations by a younger age at diagnosis and demonstrate female dominance in the whole patient group. High-resolution chest CT can help assist in differentiating PAH with PVOD/PCH*. BMPR2* variants and biallelic *EIF2AK4* variants are associated with adverse outcomes, but the survival of patients with biallelic *EIF2AK4* variants is dismal.

## Background

Pulmonary arterial hypertension (PAH) is a devastating disorder ultimately leading to increased pulmonary vascular resistance and right ventricular failure [1]. Variants in the gene encoding the bone morphogenetic protein type 2 receptor (*BMPR2*) are the most common genetic cause of PAH. Approximately 14.5–20% of idiopathic (IPAH) patients and 53.3–80% of heritable (HPAH) patients harbour variants at the *BMPR2* locus from different populations [2–5]. Patients with PAH and *BMPR2* variants present at a younger age with more severe disease and are at increased risk of death compared with those without *BMPR2* variants [3]. However, variants in *BMPR2* have also been reported in patients with histologically proven pulmonary veno-occlusive disease (PVOD) and pulmonary capillary haemangiomatosis (PCH) [6, 7], which are rare forms of PAH under the current classification [1].

A significant advance in the molecular diagnosis of PVOD/PCH was the finding of biallelic variants in the gene encoding eukaryotic translation initiation factor 2 alpha kinase 4 (*EIF2AK4*) [8, 9]. Pathogenic biallelic *EIF2AK4* variants are rarely identified (< 2%) in patients diagnosed clinically with IPAH [10, 11]. It is suggested that *EIF2AK4* variants are specific to PVOD/PCH and that finding biallelic *EIF2AK4* variants in a patient with pulmonary hypertension would be diagnostic of PVOD/PCH. Patients with PVOD/PCH have a poor prognosis and risk fatal pulmonary oedema with the use of pulmonary artery vasodilator therapies [7, 12]. Given that both *BMPR2* variant carriers and biallelic *EIF2AK4* variant carriers have poor prognoses, the different clinical phenotypes of the above gene variant carriers have not been fully clarified in all populations [11].

Previous investigators have extensively described the clinical characteristics of patients diagnosed with PAH who carry *BMPR2* variants or PVOD/PCH who carry biallelic *EIF2AK4* variants in European and American populations, but Chinese populations are not well represented in these reports [12, 13]. Ethnic background may influence the clinical characteristics of patients diagnosed with PAH or PVOD/PCH [2, 14]. Here, we report the clinical characteristics of patients diagnosed with PAH who carry *BMPR2* or biallelic *EIF2AK4* variants in a Chinese PAH population, and long-term survival was also compared.

## Methods

### Ethical approval and consent

The study was approved by the ethics committees of Fuwai Hospital (Approval No: 2017–877) and adhered to the Declaration of Helsinki. All patients recruited to the present study provided written informed consent for genetic analysis and the capture of clinical data.

### Recruitment and patients

The diagnosis of PAH was made by using pulmonary artery catheterization to confirm a mean pulmonary arterial pressure (mPAP) ≥ 25 mmHg, pulmonary capillary wedge pressure (PCWP) ≤ 15 mmHg, and pulmonary vascular resistance (PVR) > 3 Wood units. Additional clinical information (e.g., history of exposure to anorexigens) was collected, and diagnostic testing excluded associated PAH and alternative diagnoses (e.g., chronic thromboembolic pulmonary hypertension). Patients with idiopathic PAH, familial PAH and PVOD/PCH who were clinically diagnosed according to international guidelines by a specialist at Fuwai Hospital pulmonary vascular centre in China between November 2015 and June 2019 were recruited. Throughout this article, we classify patients as having idiopathic PAH or familial PAH on the basis of the absence or presence of a family history of the disease, respectively.

### Genetic analysis

The detailed genetic analysis method was previously described, and we briefly describe it here [4]. A custom-designed gene panel with 13 well-associated PAH genes was used for gene testing. This gene panel was 87 KB with 98.7% coverage of the target regions. These 13 genes include *BMPR2, KCNK3* (potassium two-pore domain channel subfamily K member 3), *CAV1* (caveolin 1), *SMAD9* (SMAD family member 9), *BMPR1B* (bone morphogenetic protein receptor type 1B), *ACVRL1* (activin A receptor-like type 1), *ENG* (endoglin), *GDF2* (growth differentiation factor 2), *SMAD4* (SMAD family member 4), *EIF2AK4, KCNA5* (potassium voltage-gated channel subfamily A member 5), *NOTCH3* (notch 3) and *TOPBP1* (DNA topoisomerase II binding protein 1). Variants with a minor allele frequency of > 0.1% in panel genes other than *EIF2AK4* were excluded as polymorphism variants, whereas *EIF2AK4* variants with a minor allele frequency of > 1% were excluded due to an autosomal-recessive inherited pattern. The remaining variants were classified into five categories according to the recommendations of the American College of Medical Genetics: benign, likely benign, unknown significance, likely pathogenic and pathogenic. Only likely pathogenic and pathogenic variants of *BMPR2* or *EIF2AK4* variants were included for further analysis.

### Clinical data collection and follow-up

Patients’ clinical records were reviewed to capture phenotypic variables from the time of diagnosis and followed-up for a median period of 22.77 months. Haemodynamic evaluation by right heart catheterization was performed at baseline in all patients. PCWP, mPAP, mixed venous oxygen saturation (SvO2) and arterial oxygen saturation (SaO2) were collected. Cardiac output (CO) was measured using the standard Fick technique. Baseline haemodynamic data and response to short-term vasodilator iloprost (20 μg for 10 min) were obtained. Vasodilator responsiveness was defined as a reduction in mPAP of at least 10 mmHg compared with baseline and attaining a level less than 40 mmHg with no reduction in CO after administration of inhaled iloprost.

Patients received follow-up every 6 months through telephone or clinic visits. The primary outcome was the composite of death or lung transplantation. Outcomes were censored if a patient was lost to follow-up or reached the end of the follow-up period. The date of PAH diagnosis was defined as the date of diagnostic right heart catheterization.

### Statistical analysis

Baseline characteristics of patients according to *BMPR2* or *EIF2AK4* variants were compared using t tests for continuous variables and χ2 tests for categorical variables. Associations of *BMPR2* or biallelic *EIF2AK4* variant status with risk of death or transplantation recorded during follow-up were assessed using Cox proportional hazards regression models. Survival curves comparing patients with *BMPR2* variants or biallelic *EIF2AK4* variants were calculated using unadjusted Kaplan-Meier estimates and compared using the log-rank test (GraphPad Software, Inc). A two-sided *p* value less than 0.05 was considered statistically significant throughout the study. Statistical analyses were performed using SPSS (version 23; IBM Corp, 2015).

## Results

### Study patients

In total, genetic analysis was performed on 232 PAH patients in the study. Fifteen patients diagnosed with associated PAH were excluded, and a total of 217 patients underwent further analysis. Of 217 PAH patients, 22 patients from 19 families exhibited confirmed familial aggregation of the disease, and 195 patients with negative family histories were classified as idiopathic PAH cases.

Predicted deleterious *BMPR2* variants were found in 12 probands (63%) with familial PAH and 41 patients (21%) with idiopathic PAH. Seven (including two siblings from one family) of the 19 patients (36.8%) with clinical diagnosis of PVOD/PCH carried biallelic *EIF2AK4* variants (1 homozygote and 6 compound heterozygotes). Six *EIF2AK4* variants were also found in 4 patients diagnosed clinically with PAH, in whom there was no clinical suspicion of PVOD/PCH (1 homozygote and 3 heterozygotes). Of the 11 biallelic *EIF2AK4* variant carriers, 3 were familial cases (2 families), 8 were sporadic cases, and no history of solvent or chemotherapy exposure was noted. Notably, no gene defects were detected in 4 patients with HPAH in our cohort, and further genetic testing for the identification of new genes potentially related to PAH is in progress.

Therefore, 45 patients carrying likely pathogenic or pathogenic *BMPR2* variants and 11 patients carrying biallelic *EIF2AK4* variants (see Supplement Table 1 & 2, some data have been reported.) with intact clinical data underwent further examination in the study.

### Clinical features of PAH carriers with *BMPR2* or biallelic *EIF2AK4* variants

Patients with a clinical diagnosis of PAH and with *BMPR2* or biallelic *EIF2AK4* variants presented with symptoms at the age of 25.57 ± 10.17 years and 31.6 ± 9.38 years, respectively. The whole group of patients showed female dominance with *BMPR2* variants and with biallelic *EIF2AK4* variants (Table 1).

**Table 1:** Demographics and clinical characteristics of patients at diagnosis with *BMPR2* and with biallelic *EIF2AK4* variants

	*BMPR2* (*N* = 45)	*EIF2AK4* (*N* = 11)	*p* value
F/M, (F%)	30/15(66.7%)	8/3(72.7%)	0.7
Age at diagnosis, y	25.57 ± 10.17	31.6 ± 9.38	0.09
BMI, kg/m^2^	21.77 ± 3.99	20.78 ± 3.49	0.49
NYHA functional class			
I-II(n/%)	21/46.7%	5/45.5%	0.94
III(n, %)	22/48.9%	5/45.5%	0.84
IV(n, %)	2/4.4%	1/9.1%	0.54
NT-proBNP, pg/ml	1806.41 ± 1466.61	1703.5 ± 1778.76	0.85
DLCO, predicted %	67.74 ± 14.63	29.88 ± 6.01	0.00*
Peak VO2, predicted %	32.56 ± 12.83	29.12 ± 9.70	0.48
6MWD, m	365.77 ± 96.11	390.56 ± 130.86	0.53
SvO2, %	64.19 ± 6.96	70.87 ± 7.34	0.02*
SaO2, %	95.11 ± 3.54	92.00 ± 2.84	0.03*
RAP, mmHg	5.11 ± 3.99	4.29 ± 2.69	0.61
mPAP, mmHg	63.62 ± 14.97	51.00 ± 4.24	0.00
PAWP, mmHg	7.15 ± 3.45	7.57 ± 2.99	0.77
PVR, Wood units	18.85 ± 6.77	12.03 ± 2.00	0.01*
CI, L/min/m2	2.39 ± 0.52	3.24 ± 1.04	0.07
Vasodilator responder	0	1/9%	–

Values are shown as mean ± SD or n (%). *BMI* body mass index, *NT-proBNP* N-terminal pro-B-type natriuretic peptide, *DLCO* diffusing lung capacity of carbon monoxide, *PeakVO2* peak oxygen consumption, *6MWD* 6 min walk distance, *SvO2* mixed venous oxygen saturation, *SaO2* arterial oxygen saturation, *RAP* right atrial pressure, *mPAP* mean pulmonary artery pressure, *PAWP* pulmonary artery wedge pressure, *PVR* pulmonary vascular resistance, *CI* cardiac index *:*p*<0.05

All patients had advanced NYHA functional class at presentation, as well as severe functional impairments. However, no significant differences in N-terminal pro-B-type natriuretic peptide (NT-proBNP) value, 6-min walk distance (6MWD) and peak oxygen consumption when expressed as percentage predicted (PeakVO2%) adjusted for age, height, and sex were found between the two groups. Notably, patients with biallelic *EIF2AK4* variants exhibited a reduced diffusing lung capacity of carbon monoxide (DLCO, [29.88 ± 6.01]% predicted) compared with *BMPR2* variant carriers ([67.74 ± 14.63]% predicted; *p* = 0.00).

Right heart catheterization at diagnosis showed severe pre-capillary pulmonary hypertension in both groups. Haemodynamic variables, including SvO2, PVR and cardiac index (CI), were more severely impaired in carriers with *BMPR2* variants than in carriers with biallelic *EIF2AK4* variants (Table 1). Acute vasodilator testing was performed in both groups without acute pulmonary oedema complications. One (9%) of 11 carriers of biallelic *EIF2AK4* variant carriers fulfilled the haemodynamic criteria for significant acute vasoreactivity, while none of the *BMPR2* carriers responded to acute vasodilators (Table 1).

### CT features of PAH carriers with *BMPR2* or biallelic *EIF2AK4* variants

High-resolution Computed Tomography (CT) of the chest was available in 38 *BMPR2* variant carriers and 11 biallelic *EIF2AK4* variant carriers. Centrilobular ground glass opacification, interlobular septal thickening and mediastinal lymphadenopathy are considered suggestive signs of PVOD/PCH. The characteristic CT signs were more prominent in biallelic *EIF2AK4* variant carriers than in *BMPR2* variant carriers. Specifically, we found centrilobular ground glass opacification in 10 (90.9%), interlobular septal thickening in 9 (81.8%), and mediastinal lymphadenopathy in 5 (45.5%) patients carrying biallelic *EIF2AK4* variants (Table 2). More than 80% of biallelic *EIF2AK4* variant carriers presented with at least two of these radiological abnormalities at diagnosis, and 4 (36.4%) displayed all three radiological abnormalities. In contrast, such characteristic CT features were less commonly presented in patients carrying *BMPR2* variants. Twenty-four (63.2%) PAH patients with *BMPR2* variants demonstrated no radiological abnormalities on high-resolution CT. However, three (7.9%) *BMPR2* variant carriers presented with all three radiological abnormalities, whose percentage was significantly lower compared with 36.4% of patients with biallelic *EIF2AK4* variants, *p* = 0.0017.

**Table 2:** Radiological Features of Patients with *BMPR2* mutations or biallelic *EIF2AK4* variants

	*BMPR2* (*N* = 38) n(%)	*EIF2AK4* (*N* = 11) n(%)	*p* value
Centrilobular ground glass opacification	13(34.2)	10(90.9)	0.001*
Interlobular septal thickening	11(28.9)	9(81.8)	0.002*
Mediastinal lymphadenopathy	4(10.5)	5(45.5)	0.008*
Three signs	3(7.9)	4(36.4)	0.017*
Two signs	8(21.1)	5(45.5)	0.11
One sign	3(7.9)	2(18.2)	0.32
None	24(63.2)	0	–

Abbreviations see Table 1

### Survival of patients with *BMPR2* or biallelic *EIF2AK4* variants

Most of the patients with *BMPR2* variants received mono- or combined specific medical therapy for PAH (see Table 3), while 18.2% of patients with *EIF2AK4* variant carriers received no PAH-specific therapy.

**Table 3:** Specific Medical therapy for PAH with *BMPR2* mutations or biallelic *EIF2AK4* variants

	*BMPR2* (*N* = 45)	*EIF2AK4* (*N* = 11)
ERA monotherapy	5(11.1)	0
PDE5 inhibitor monotherapy	21(46.7)	5(45.5)
Prostacyclin derivative monotherapy	1(2.2)	1(9.1)
ERA plus PDE5	14(31.3)	3(27.3)
ERA plus prostacyclin derivative	1(2.2)	0
PDE5 inhibitor plus prostacyclin derivative	1(2.2)	0
ERA plus PDE5 inhibitor plus prostacyclin derivative	1(2.2)	0
No specific PAH therapy	1(2.2)	2(18.2)

*ERA* endothelin receptor antagonist, *PDE5* phosphoriesterase type 5

**Fig. 1:**
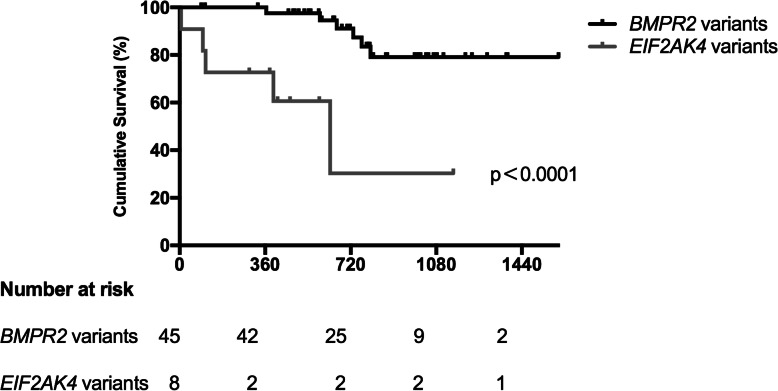
Kaplan-Meier survival curves according to *BMPR2* and biallelic *EIF2AK4* variants status (*p*<0.0001)

Patients diagnosed clinically as having PAH with biallelic *EIF2AK4* variants exhibited a shorter survival time from diagnosis compared with the *BMPR2* variant carriers (*p* < 0.0001 for log-rank test, Fig. 1). *BMPR2* or biallelic *EIF2AK4* variants, low DLCO, low Peak VO2% and reduced 6MWD were associated with an increased risk for death or lung transplant (Table 4). However, no significant effects of haemodynamic measurements on survival were observed in the present study.

**Table 4:** Proportion of excess risk mediated by genetic status and clinical variables at diagnosis

Death or transplantation	HR (95% CI)	*p* value
*BMPR2* or biallelic *EIF2AK4* variants	0.114(0.032–0.404)	0.001*
Adjusted for DLCO	0.035(0.001–1.032)	0.05
Adjusted for DLCO, Peak VO2%, 6MWD	0.001(0.000–0.179)	0.011*
DLCO	0.958(0.919–0.999)	0.042*
Adjusted for *BMPR2* or biallelic *EIF2AK4* variants	1.024(0.954–1.09)	0.509
Adjusted for Peak VO2%, 6MWD, *BMPR2* or *EIF2AK4* mutations	1.158(1.002–1.339)	0.047*
Peak VO2%	0.89(0.819–0.987)	0.025*
Adjusted for *BMPR2* or biallelic *EIF2AK4* variants	0.883(0.803–0.971)	0.011*
Adjusted for DLCO, 6MWD, *BMPR2* or *EIF2AK4* mutations	0.725(0.505–1.039)	0.08
6MWD	0.992(0.984–1)	0.04*
Adjusted for *BMPR2* or biallelic *EIF2AK4* variants	0.992(0.985–0.999)	0.018*
Adjusted for DLCO, Peak VO2%, *BMPR2* or biallelic *EIF2AK4* variants	1.00(0.988–1.013)	0.975
Cardiac Index	0.778(0.285–2.122)	0.624
Pulmonary vascular resistance	1(0.999–1.001)	0.429
Mean pulmonary arterial pressure	1.013(0.971–1.057)	0.55
SvO2	1.009(0.923–1.1037)	0.841

*HR* Hazard ratios, other abbreviations see Table 1

## Discussion

Substantial progress has been made in understanding the clinical characteristics based on various PAH genetic backgrounds during the past decade from different populations [2–4, 10–12, 14–16]. The present study provides data for a Chinese population describing the clinical and radiological features of PAH patients carrying either pathogenic *BMPR2* variants or pathogenic biallelic *EIF2AK4* variants.

Previous studies have reported that *BMPR2* variants are the most common genetic cause of PAH [1, 3, 15]. As expected, pathogenic *BMPR2* variants were most commonly found in patients with a clinical diagnosis of HPAH or idiopathic PAH in the present study. In addition, we identified a high frequency of biallelic *EIF2AK4* variants in patients with a clinical presentation of PVOD/PCH. However, we also found biallelic *EIF2AK4* variants in patients with a clinical diagnosis of PAH, which was consistent with other observations [11]. Therefore, gene testing for *BMPR2* variant detection is important in all eligible PAH patients. However, for a PAH patient with clinical suspicion of PVOD/PCH, biallelic *EIF2AK4* variant testing is recommended, regardless of familial history. The discovery of biallelic *EIF2AK4* variants in PVOD/PCH raised the possibility of rapid molecular diagnosis in patients with familial and sporadic PVOD/PCH. In the present study, the presence of biallelic *EIF2AK4* variants was associated with a poor prognosis compared to PAH patients with *BMPR2* variants. Therefore, genetic testing can help identify patients with pathogenic *EIF2AK4* variants early and may facilitate timely PVOD/PCH diagnosis and referral for lung transplantation before their conditions deteriorate.

The presence of *BMPR2* variants or biallelic *EIF2AK4* variants possesses similar demographic characteristics and clinical presentations in the present study. As we noted, all patients presented with clinical, functional and haemodynamic impairments regardless of whether they were *BMPR2* variant carriers or *EIF2AK4* variant carriers. However, in contrast to previous studies [11, 12], patients with *BMPR2* or biallelic *EIF2AK4* variants were at a younger age when diagnosed in our study, although clinical and haemodynamic inclusion criteria were similar. It is interesting to note that in a previous study from China, the authors also found the mean age of patients was younger in a Chinese Han cohort than the age of patients with IPAH or HPAH in other populations [2]. Although it is not easy to speculate the reason for such a difference, it may possibly be ascribed to an increasing pressure of geographic- and population-specific factors.

Another striking result is that the predominant proportion of females affected by *EIF2AK4* variants contrasts with results reported by Montani et al., who showed an equal number of men and women affected by *EIF2AK4* variants from different populations [12]. We acknowledge that it is difficult to speculate the reason for the diversity based on such a small population. However, possible explanations include increased representation of ethnic groups with a higher female: male ratio in Chinese PAH patients with biallelic *EIF2AK4* variants. This may also be ascribed to other race-specific factors and environmental backgrounds. However, further large-scale investigation is needed to verify these observations.

Moreover, it is worth noting that patients with biallelic *EIF2AK4* variants demonstrated a significantly reduced DLCO compared with patients with *BMPR2* variants. The distinctive CT features with centrilobular ground glass opacification and interlobular septal thickening indicate thickening of the blood-gas barrier, which may lead to widespread reduction in alveolar gas exchange and thus reduction in DLCO in biallelic *EIF2AK4* variant carriers.

It is generally recognized that high-resolution CT imaging is a useful noninvasive method to assist in the diagnosis of suspected PVOD/PCH and direct the patient towards specialist review [17]. In keeping with previous studies, we found an increased prevalence of centrilobular ground glass opacification, interlobular septal thickening and mediastinal lymphadenopathy in patients with biallelic *EIF2AK4* variants. We also noted that approximately one-third of biallelic *EIF2AK4* variant carriers demonstrated all three distinctive CT features, indicating differing radiological features of similar genetic backgrounds in various individuals. In addition, we found some patients with *BMPR2* variants revealing diffuse ground glass opacity with thickening interlobular septa in high-resolution CT, which might be misdiagnosed as PVOD/PCH. This was similar to a previous study, in which a radiological suspicion of PVOD/PCH was presented in 71% of those with PVOD/PCH, 57% of patients with a clinical diagnosis of PAH and biallelic *EIF2AK4* variants, 14% of patients with PAH with no variant, and 5% of those with *BMPR2* variants [11]. What differed from the previous study was that centrilobular ground glass opacification was less frequent; however, interlobular septal thickening abnormalities were more commonly present among PAH patients with *BMPR2* variants in our observation. In other words, radiologic features at the time of diagnosis could not accurately determine the exact diagnosis and underlying genotype.

According to 2015 ERS/ESC guidelines, low CI and SvO_2_ are associated with poor outcomes [1]. However, in the present study, we found no association between hemodynamic variables and primary outcomes. This might be ascribed to the following possible reasons. First, the sample size of the study was relatively small, but continuing patients are still enrolling. Besides, we used variables at the time of diagnosis when patients might have normal CI and/or SvO2 and we don’t have any follow up information on hemodynamic. However, we noted lower Peak VO2% and shorten 6MWD were associated with poor survival in our study. All these observations might imply exercise capacity decrease before hemodynamic deteriorating.

### Limitation

There are some limitations to our study. On the one hand, the sample size was relatively small, which might inevitably weaken the force of our conclusions. However, PAHs are orphan diseases, and we are continuing to enroll new PAH patients to verify the present findings. In addition, we compared PAH patients with *BMPR2* or biallelic *EIF2AK4* variants without pathological examination. Current guidelines indicate that a diagnosis of heritable PVOD/PCH can be made by identifying pathogenic biallelic *EIF2AK4* variants without necessitating lung biopsy [9, 18], which may pose a substantial risk for morbidity or mortality for a patient with severe pulmonary hypertension and/or right ventricular failure. Therefore, we used strict clinical criteria, including high-resolution CT and DLCO, to analyse the phenotypes of these PAHs.

## Conclusion

Our study reports the clinical and radiological features together with survival in a Chinese cohort diagnosed with PAH, with the genetic background of *BMPR2* and biallelic *EIF2AK4* variants. The overall clinical characteristics of PAH patients with *BMPR2* and biallelic *EIF2AK4* variants in Chinese patients were similar to those of other populations. However, a few characteristics were noteworthy: Chinese PAH patients presented with a younger age at diagnosis compared with other populations. In addition, a female predominance among PAH patients with biallelic *EIF2AK4* variants was also noted. All of these discrepancies may be due to geographic and race-specific factors, and some environmental elements may also play a role. However, further investigations are still warranted. High-resolution CT reveals a high incidence of radiological abnormalities in PAH patients with biallelic *EIF2AK4* variants. However, similar radiological abnormalities can also be found in some *BMPR2* variant carriers. *BMPR2* variants and biallelic *EIF2AK4* variants are associated with adverse outcomes, but the survival of patients with biallelic *EIF2AK4* variants is dismal. As a result, genetic testing with high-resolution CT may assist in the identification of PVOD/PCH in a timely manner and in referring for lung transplantation before their condition deteriorates.

## Supplementary information

**Additional file 1: Table S1** Pathogenic/Likely Pathogenic *BMPR2* variants. **Table S2** Pathogenic/Likely Pathogenic biallelic *EIF2AK4* mutations

## Data Availability

The datasets used and/or analyzed during the current study are available from the corresponding author on reasonable request.

## Abbreviations

PAH: Pulmonary arterial hypertension
BMPR2: Bone morphogenetic protein type 2 receptor
IPAH: Idiopathic pulmonary arterial hypertension
HPAH: Heritable pulmonary arterial hypertension
PVOD/ PCH: Pulmonary veno-occlusive disease/ pulmonary capillary haemangiomatosis
*EIF2AK4*: Eukaryotic translation initiation factor 2 alpha kinase 4
KCNK3: Potassium two-pore domain channel subfamily K member 3
CAV1: Caveolin 1
SMAD9: SMAD family member 9
BMPR1B: Bone morphogenetic protein receptor type 1B
ACVRL1: Activin A receptor-like type 1
ENG: Endoglin
GDF2: Growth differentiation factor 2
SMAD4: SMAD family member 4
KCNA5: Potassium voltage-gated channel subfamily A member 5
NOTCH3: Notch 3
TOPBP1: DNA Topoisomerase II binding protein 1
mPAP: Mean pulmonary arterial pressure
PCWP: Pulmonary capillary wedge pressure
PVR: Pulmonary vascular resistance
SvO2: Mixed venous oxygen saturation
SaO2: Arterial oxygen saturation
CO: Cardiac output
NT-proBNP: N-terminal pro-B-type natriuretic peptide
6MWD: 6-min walk distance
DLOC: Diffusing lung capacity of carbon monoxide
CI: Cardiac index
CT: computed tomography
