# Supplementary material for: Clinical characteristics and survival of Chinese patients diagnosed with pulmonary arterial hypertension who carry BMPR2 or EIF2KAK4 variants
Source: BMC Pulm Med. 2020 May 29;20:150. doi: 10.1186/s12890-020-01179-7 (PMC7257189; doi:10.1186/s12890-020-01179-7)
Supplement: Supplementary file 1 — Additional file 1: Table S1 Pathogenic/Likely Pathogenic BMPR2 variants. Table S2 Pathogenic/Likely Pathogenic biallelic EIF2AK4 mutations [file 12890_2020_1179_MOESM1_ESM.docx]

Supplementary Table 1. Pathogenic/Likely Pathogenic *BMPR2* variants

| Patients ID | Nucleotide change | Protein change | MLPA |
| --- | --- | --- | --- |
| PAH1 | c.551dup | p.His184fs |  |
| PAH10 | c.796_799del | p.Arg266fs |  |
| PAH17 | c.631C＞T | p.Arg211Ter |  |
| PAH22 |  |  | Ex1 del, het |
| PAH27 | c.338dup | p.Tyr113Ter |  |
| PAH31 | c.439C＞T | p.Arg147Ter |  |
| PAH34 | c.705del | p.Asn236fs |  |
| PAH36 |  |  | Ex4 del, het |
| PAH44 |  |  | The whole gene del, het |
| PAH47 | c.2569del | p.Arg857fs |  |
| PAH51 | c.1372C＞T | p.Gln458Ter |  |
| PAH52 |  |  | Ex11-12 del, het |
| PAH60 |  |  | Ex1 del, het |
| PAH67 | c.944T＞C | p.Leu315Pro |  |
| PAH75 | c.1089del | p.Val364fs |  |
| PAH76 | c.47G＞A | p.Trp16Ter |  |
| PAH84 |  |  | Ex6-7 del, het |
| PAH98 | c.631C＞T | p.Arg211Ter |  |
| PAH109 |  |  | The whole gene del, het |
| PAH117 | c.178T＞C | p.Cys60Arg |  |
| PAH138 | c.1249insAT | p.Phe417fs |  |
| PAH143 | c.1789C＞T | p.Arg597Ter |  |
| PAH145 | c.1862C＞ | p.Thr621Met |  |
| PAH150 | c.994C＞T | p.Arg332Ter |  |
| PAH153 | c.2617C＞T | p.Arg873Ter |  |
| PAH158 |  |  | Ex1 del, het |
| PAH159 | c.2470C＞T | p.Gln824Ter |  |
| PAH163 | c.2522del | p.His841fs |  |
| PAH166 | c.2810_2831dup | p.Thr946Ter |  |
| PAH174 |  |  | Ex8-9 del, het |
| PAH176 | c.631C＞T | p.Arg211Ter |  |
| PAH177 | c.631C＞T | p.Arg211Ter |  |
| PAH178 | c.714_715del | p.Asn239fs |  |
| PAH183 | c.852+ 3A＞C |  |  |
| PAH184 | c.439C＞T | p.Arg147Ter |  |
| PAH187 | c.1090del | p.Val364fs |  |
| PAH190 |  |  | Ex1 del, het |
| PAH192 | c. 2269dup | p.Ser757fs |  |
| PAH195 | c.1161del | p.Leu388Ter |  |
| PAH199 | c.631C＞T | p.Arg211Ter |  |
| PAH210 | c.2267del | p.Thr756fs |  |
| PAH212 | c.1241G＞A | p.Trp414Ter |  |
| PAH231 | c.2617C＞T | p.Arg873Ter |  |
| PAH236 | c.1279del | p.Glu427fs |  |
| PAH240 | c.1231_1244del | p.Leu411fs |  |

Supplementary Table 2. Pathogenic/Likely Pathogenic biallelic *EIF2AK4* mutations

| Patients ID | Variant (1) | Variant (2) |
| --- | --- | --- |
| PAH3 | c.2403+1G＞A, het | c.2632-1G＞A, het |
| PAH48 | c.2965C＞T:p.Arg989Trp, het | c.4724T＞C:p.Leu1575Pro, het |
| PAH169 | c.170del:p.Asn57fs, het | c.4460-1G＞A, het |
| PAH197 | c.597+1G＞A, het | c.2965C＞T:p.Arg989Trp, het |
| PAH202 | c.989_990del:p.Lys330fs, hom |  |
| PAH215 | c.1753C＞T:p.Arg585Ter, het | c.1628C＞T:p.Pro543Leu, het |
| PAH227 | c.4833_4836dup:p.Gln1613fs, het | c.1804G＞A:p.Gly602Arg, het |
| PAH237 | c.3460A＞T:p.Lys1154Ter, het | c.4736T＞C:p.Leu1579Pro, het |
| PAH238 | c.4833_4836dup:p.Gln1613fs, het | c.1942A>T:p.Ile648Phe, het |
| PAH241 | c.3964C＞T:p.Gln1322Ter, hom |  |
